# Targeting TLK2 with antisense oligonucleotides as a new strategy in acute myeloid leukemia

**DOI:** 10.3389/fonc.2026.1659341

**Published:** 2026-03-09

**Authors:** Hsin-Yun Lin, Sokchea Khou, Evan Lind, Katia G. de Oliveira Rebola, Ariel Dean, Haijiao Zhang, Angelica Tolentino, Hadi Maazi, Alexey Revenko, Anupriya Agarwal

**Affiliations:** 1 Division of Oncological Sciences, Oregon Health & Science University, Portland, OR, United States; 2 Department of Cell, Developmental, and Cancer Biology, Oregon Health & Science University, Portland, OR, United States; 3 Department of Molecular and Medical Genetics, Oregon Health & Science University, Portland, OR, United States; 4 Knight Cancer Institute, Oregon Health & Science University, Portland, OR, United States; 5 Department of Molecular Microbiology and Immunology, Oregon Health & Science University, Portland, OR, United States; 6 Ionis Pharmaceuticals, Inc., Carlsbad, CA, United States

**Keywords:** AML, antisense oligonucleotide, drug resistance, gilteritinib, Tlk2

## Abstract

**Introduction:**

Tousled-like kinase 2 (TLK2) is a serine/threonine kinase that plays a role in DNA replication, chromatin remodeling, and DNA damage response. TLK2 has been implicated in the pathogenesis of various types of cancer, including breast cancer, glioblastoma, and acute myeloid leukemia (AML). However, no potent and selective TLK2 inhibitors have been developed.

**Methods:**

We evaluated the efficacy of a human TLK2 antisense oligonucleotide (ASO) alone and in combination with gilteritinib in AML cell lines. To assess in vivo efficacy and toxicity, we administered a mouse TLK2 ASO alone or in combination with gilteritinib in a murine model of AML.

**Results:**

TLK2 ASO treatment resulted in a dose-dependent reduction of TLK2 mRNA levels and decreased cell viability in FLT3-mutant AML cell lines, with enhanced cytotoxicity observed when combined with gilteritinib. In a murine AML model, TLK2 ASO achieved approximately 50% knockdown efficiency. Both TLK2 ASO alone and in combination with gilteritinib significantly reduced spleen size, leukemia burden, and bone marrow progenitor cell populations. The treatment was generally well tolerated, with only minimal toxicity observed.

**Discussion:**

This study demonstrates that TLK2 ASO is a promising therapeutic strategy for AML, particularly in combination with FLT3 inhibition, and may apply to other TLK2-driven cancers. Future efforts should focus on improving ASO delivery and knockdown efficiency to maximize therapeutic benefit.

## Introduction

Acute myeloid leukemia (AML) is one of the most aggressive forms of hematological malignancies, characterized by uncontrolled proliferation and abnormally differentiated myeloid precursor cells. Despite advances in supportive care and targeted therapies, outcomes for AML patients have improved only slightly over four decades (1), largely due to significant heterogeneity in the molecular abnormalities driving AML (2). Selective inhibitors targeting many pathways affected by these genetic alterations have been developed; however, their successful translation into the clinic is impeded by disease heterogeneity and drug resistance (3, 4).

We recently demonstrated that Tousled-like kinase 2 (TLK2) overexpression contributes to the survival and proliferation of AML cells with various genetic subtypes, and targeting TLK2 may represent a promising therapeutic strategy for AML (5). TLK2 is a serine/threonine kinase that plays roles in DNA replication, chromatin remodeling, and DNA damage response (6, 7). TLK2 is regulated by dimerization and autophosphorylation, and the crystal structure of TLK2 reveals mechanisms of activation and provides a basis for structure-guided inhibitor design (8). TLK2 has been implicated in the pathogenesis of various types of cancer, including breast cancer, glioblastoma, and AML (5, 9, 10). However, despite these findings, no potent and selective TLK2 inhibitors are available yet (11).

One major challenge in targeting TLK2 with small molecules is its close sequence homology with its paralog TLK1, particularly within the C-terminal kinase domain (11). Recently, two PKC inhibitors, Go6983 and GF109203X, were identified using the kinase profiling to possess selectivity against TLK2. While they showed *in vitro* efficacy at high doses in breast cancer MCF-7 cells, they have not been tested *in vivo* due to their off-target effects (10). Further, recent developments based on indirubin derivatives still inhibited both TLK2 and TLK1, underscoring the difficulty of achieving TLK2 specificity with conventional kinase inhibitors (12). Such off-target effects also complicate the interpretation to dissect which TLK is responsible for the observed phenotypes, suggesting a need for specific TLK1 or TLK2 targeting strategies.

Interestingly, DepMap analysis indicates that AML cell lines show significant dependency on TLK2 across various genotypes, while TLK1 dependency is variable. This is consistent with our previous study, in which combined knockdown of TLK1 and TLK2 provided only a modest benefit in reducing the proliferation and growth of AML cells compared to TLK2 knockdown alone (5). Furthermore, broad inhibition of both TLK kinases may increase the risk of toxicity, given their shared roles in DNA replication and the DNA damage response in normal tissues. For this reason, a TLK2-selective therapeutic approach is currently favored to achieve efficacy while minimizing off-target effects.

Antisense oligonucleotides (ASOs) have emerged as a promising therapeutic approach for treating various cancers (13). ASOs are short, single-stranded DNA molecules that bind complementary RNA sequences to modulate gene expression. Depending on their design, ASOs can induce RNA degradation, block translation, or alter splicing (14). Gapmer ASOs contain chemically modified “wings” flanking a central DNA “gap”; the modified wings increase stability, while the DNA gap recruits RNase H to cleave the target mRNA within the RNA–DNA duplex. In recent years, significant progress has been made to improve the biologic activity, specificity, and toxicity of ASOs. This has enabled targeting of genes previously considered “undruggable, “ including transcription factors and regulatory proteins, using ASOs, as a valuable clinical strategy for treating various diseases (15, 16). Several ASO-based therapies, such as those for spinal muscular atrophy (17), have already been used in clinical settings. In AML, ASOs can target genes involved in leukemic cell proliferation, differentiation, and survival, such as BCL-2, STAT3, and FLT3 (18–20). Several clinical studies have also shown the efficacy of ASOs in reducing leukemic cell viability, inducing apoptosis, and improving overall survival in AML patients (21). In this study, we demonstrated the feasibility of specifically targeting TLK2 using an ASO-based strategy. Additionally, we tested the efficacy of TLK2 ASO in combination with the, inhibitor of FLT3 mutations in which are present in about one-third of AML patients. We showed that combining TLK2 ASO with FLT3 inhibitor gilteritinib effectively suppressed leukemic growth *in vitro* and *in vivo*.

## Materials and methods

### Cell lines

All cell lines were confirmed as negative for mycoplasma. The MOLM-14 cell line was cultured in RPMI 1640 (Gibco) media supplemented with 10% fetal bovine serum (FBS), 2 mM L-glutamine, 100 U/ml penicillin, and 100 µg/mL streptomycin. The MONOMAC-1 cell line was cultured in the same media and supplemented with non-essential amino acids and sodium pyruvate. Both cell lines were cultured at 37°C and 5% CO2.

### Antisense oligonucleotides

Non-targeting (NT– Mouse/Human 792169) and Tousled Like Kinase 2 (TLK2 – Mouse 1545612 and Human 1523195) antisense oligonucleotides (ASOs) were provided by IONIS Pharmaceutical, Inc. The human TLK2 ASO (1523195) and mouse TLK2 ASO (1545612) are aligned exclusively to TLK2 in their respective genomes, with up to four mismatches and a minimum of 12 contiguous matching bases. Each ASO evaluated in this study was a 16-mer gapmer constructed with a backbone of phosphorothioate internucleoside linkages. To enhance target affinity and provide resistance to cellular nucleases, the first three and last three positions were modified with 2′–4′ constrained ethyl (cEt) ribonucleotides. The central region consisted of 10 deoxynucleotides, enabling RNase H1 to recognize and cleave the complementary RNA strand within the ASO: RNA duplex. Additionally, cytosine residues were 5-methylated to improve hybridization properties and reduce pro-inflammatory potential (22).

### Cell viability assays

Three replicates of MOLM-14 cells (500 cells/well) and MONOMAC-1 (1, 000 cells/well) were plated in 384-well plates and pre-treated for 4 days with either PBS, 1, 2, and 5 μM of NT or 1, 2, and 5 μM TLK2 ASOs followed by 3 days of incubation with a 7 point concentration gradient ranging from 2 to 20 nM of gilteritinib (Selleck Chem). MTS reagent (CellTiter96 Aqueous One; Promega) was added, and the optical density was measured at 490 nm. Raw absorbance values were adjusted to a reference blank value, normalized to PBS controls, and presented as fold differences to determine cell viability. IC_50s_ were calculated by nonlinear regression in GraphPad Prism 10, fitting normalized cell viability to four-parameter dose-response curves. Synergy scores of drug combinations were calculated using Combenefit and SynergyFinder software (23–25).

### Mice

All animal studies were approved by the OHSU Institutional Animal Care and Use Committee (IACUC). Animals were housed in OHSU’s semi-conventional facility and cared for according to OHSU standard guidelines. For genotyping, tail tips were collected prior to transplantation and analyzed by quantitative real-time PCR (Transnetyx).

### Quantitative PCR

RNA extraction was performed using the RNeasy mini kit (Qiagen) per manufacturer’s instructions, and RNA yield was quantified by a Nanodrop 1000 Spectrophotometer (ND-1000). RNA was converted into cDNA using the High-Capacity cDNA Reverse Transcription Kit (Thermo Fisher Scientific). Quantitative PCR was performed using Platinum SYBR Green qPCR SuperMix (Thermo Fisher Scientific), and the probes are indicated indicated in **Table 1**. qPCR cycles were run on the QuantStudio 7 Flex System with standard cycle parameters.

**Table 1:** qPCR primer sequences.

Human TLK2	Primer 1: 5’-CTGAACCATATGAAACTAGCCAAG-3’ Primer 2: 5’-AGCACTGCCATCTAAACCATAG-3’
Human TLK1	Primer 1: 5’-GCAACTCCAGTAAATCTAGCTTCC-3’ Primer 2: 5’-GTCCCTGCTGAATCACACG -3’
Human GAPDH	Primer 1: 5’-TCCTGCACCACCAACTGCTTAG-3’ Primer 2: 5’-GGCATGGACTGTGGTCATGAGT-3’
Mouse TLK2	Primer 1: 5’-CGACTTGACACAGAGCAGTT-3’ Primer 2: 5’-TATTGCCAGAACCCGTAGAAG-3’
Mouse GAPDH	Primer 1: 5’-AATGGTGAAGGTCGGTGTG-3’ Primer 2: 5’-GTGGAGTCATACTGGAACATGTAG-3’

### Western blot analysis

Whole-cell extracts were prepared by lysing cells for 20 min on ice in lysis buffer (Cell Signaling Technology) supplemented with a complete mini protease inhibitor cocktail tablet (Roche), phosphatase inhibitor (Sigma-Aldrich), and phenylmethylsulphonyl fluoride (PMSF). Protein concentrations were quantified and normalized by a bicinchoninic acid (BCA) assay (Thermo Fisher Scientific). Lysates were separated through SDS polyacrylamide gels (4-12%) and transferred to a nitrocellulose membrane (Bio-Rad). Membranes were blocked with 5% BSA in 0.1% Tween20 in 1x PBS (PBS-T) for 1 hr at room temperature, followed by incubation with primary antibodies diluted in 5% BSA in PBST overnight at 4 °C included: TLK2 (#A301-257A, Bethyl Laboratories); Phospho-Stat5 (Tyr694) (#9351, Cell Signaling Technology); Phospho-FLT3 (Tyr589/591) (#3464, Cell Signaling Technology); Actin (#MAB1501, EMD Millipore). The membrane was washed 3x in TBST before incubation with appropriate HRP-conjugated secondary antibodies for 1 h. Protein levels were visualized using ECL substrate (Bio-Rad) on a ChemiDoc MP Imaging System (Bio-Rad) with the Image Lab Software.

### Bone marrow transplantation and treatment

Bone marrow cells were isolated from bones of 95 to 100-weeks old *LysM-Cre Tet2^+/-^ Flt3^ITD^* donor mice. Lineage-negative cells were enriched using a Direct Lineage Cell Depletion Kit (Miltenyi Biotech) and pre-stimulated at 37 °C overnight in DMEM supplemented with 1% Pen/Strep, 1X L-glutamine, 15% FBS, 15% WEHI-3B, 7 ng/mL murine IL-3, 12 ng/mL IL-6, and 56 ng/mL SCF. 1e6 lineage negative cells and supporting cells were retro-orbitally injected into 6-weeks old BoyJ CD45.1 recipient mice (Jackson Lab #002014) which were lethally irradiated with two doses of 500 and 450 cGy 4 hours apart. Mice were maintained on a soft food diet and antibiotic water for 2 weeks and were carefully monitored daily for signs of disease. Engraftment was confirmed 3 weeks post-bone marrow transplantation in recipient mice. Disease progression was monitored weekly by complete blood count using Heska Vet ABC blood analyzer (Scil Animal Care Company) and by CD45.1^+^, CD45.2^+^, and CD11b^+^ percentage using BD LSRFortessa Cell Analyzer (BD Bioscience). From 14 days post-bone marrow transplant until week 6, each recipient mouse was treated daily with 25 mg/ml gilteritinib orally and 30 mpk/mouse of either NT or TLK2 ASOs subcutaneously (210 mpk/mouse weekly). From week 7 to 13, 30 mg/ml gilteritinib and either 30 mpk/mouse of NT or TLK2 ASO daily (210 mpk/mouse weekly). From week 14 to 18, 30 mg/ml gilteritinib and 50 mpk/mouse of either NT or TLK2 ASOs 5 days per week (250 mpk/mouse weekly). Morbid mice or mice that lost more than 20% of weight were euthanized for further analysis. Bone marrow and spleen cells were stained with CD11b-APC, CD19-APC eFlour780, CD3-PE-Cy7, CD45-PE, Ter119-eFlour450, Ly6g/Ly6c-BV605, CD34-Alexa-700, cKit-APC-eFlour 780, Sca1-Pac Blue, FcGamma-BV605, and FVS510-viability for flow cytometry analysis.

### Ultrasound imaging

All ultrasound imaging was performed using the Vevo^®^ 2100 system. Mice were anesthetized with continuous isoflurane. The abdominal areas were shaved, and any residual hair was removed using depilatory cream. Mice were positioned supine on a heated imaging platform to maintain body temperature, and ultrasound gel was applied to the abdominal area to facilitate signal transmission. Two-dimensional (2D) B-mode images of the spleen were acquired in longitudinal planes. Spleen volumes were obtained from the integrated standard echocardiography volume calculation in the software. All imaging parameters were maintained consistent across animals.

### Plasma chemistry

Blood samples were obtained from mice treated with control ASO, TLK2 ASO, gilteritinib, or TLK2 ASO in combination with gilteritinib. The plasma was separated by centrifugation and ALT, AST, blood urea nitrogen, bilirubin, and albumin were measured using Beckman Coulter chemistry analyzer AU480 according to the manufacturer’s protocols at Ionis Pharmaceuticals.

### Colony formation assays

2, 500 lineage-negative bone marrow cells per well from *LysM-Cre Flt3^ITD^Tet2^+/-^* mouse were plated in methylcellulose-based media (M03434) and treated with 10 µM NT ASO or TLK2 ASO, either alone or in combination with 200 nM gilteritinib. Colonies were counted on day 6.

### Statistical analysis

ASO sensitivity data for cell lines were represented as percentage viability across a concentration range. The statistical analyses used for the specific figures are indicated. In all cases, p-value < 0.05 was considered significant.

## Results

**Figure 1:**
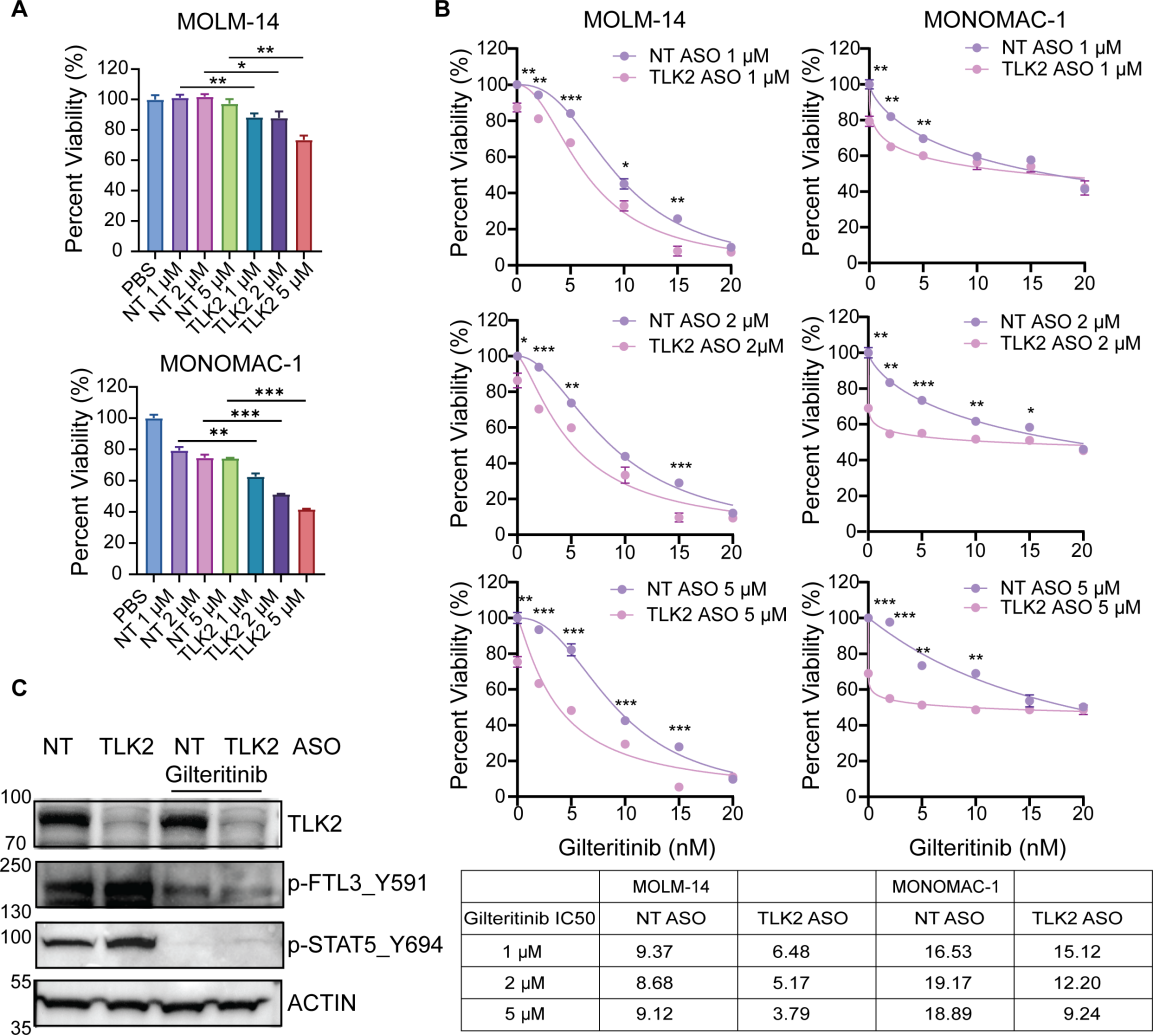
TLK2 ASO alone and in combination with gilteritinib decreases viability in AML cell lines. **(A)** MOLM-14 and MONOMAC-1 cells were treated with TLK2 ASO with a concentration gradient for 7 days, respectively. Cell viability was measured using MTS assay. **(B)** Within the same experiment (panel **A**), MOLM-14 and MONOMAC-1 cells were pre-treated with TLK2 ASO for 4 days and then treated with a FLT3 inhibitor gilteritinib with a concentration gradient for 3 days. Cell viability was measured using MTS assay. IC_50_s were calculated by nonlinear regression in GraphPad Prism 10, fitting normalized cell viability to four-parameter dose-response curves. **(C)** Immunoblot analysis of signaling in MOLM-14 cells treated with 5 µM NT or TLK ASO, in combination with 20 nM gilteritinib. Statistical significance in panels A and B (*p < 0.05, **p < 0.01, ***p < 0.001) as determined by student t-test.

We have demonstrated that TLK2 is highly expressed in AML cells (5), and genetic targeting of TLK2 reduces AML cell growth *in vitro* and *in vivo*, suggesting that TLK2 is a potential therapeutic target. Inhibition of TLK2 has been challenging due to the lack of specific small molecule inhibitors and limited *in vivo* data. Therefore, we developed a novel antisense oligonucleotide sequence that targets the human *TLK2* gene and assessed its efficacy. We focused on testing its efficacy using FLT3 mutation-positive cells, considering FLT3 mutations are found in 1/3 of AML patients. Specifically, we used two *FLT3*-mutation-positive cell lines with diverse genetic properties. MOLM-14 cells harbor the common Flt3 ^ITD^ mutation, which is present in 30% of AML patients. MONOMAC-1 cells harbor the FLT3 V592A and TP53 mutations. These two cell lines also have distinct cytogenetic backgrounds (26). Non-targeting (NT) ASO (792169) and human TLK2 ASO (1523195) were delivered to AML cells by free uptake. The concentration range for ASOs testing in the cell viability assay was selected based on prior studies using hematopoietic cells (19). Three replicates of MOLM-14 cells (500 cells/well) and MONOMAC-1 (1, 000 cells/well) were plated in 384-well plates and pre-treated for 4 days with either PBS, 1, 2, and 5 μM of NT or 1, 2, and 5 μM TLK2 ASOs followed by 3 days of incubation with a 7 point concentration gradient ranging from 2 to 20 nM of gilteritinib. TLK2 ASO reduced TLK2 mRNA levels in MOLM-14 cells at 1 and 2 μM and MONOMAC-1 cells at 1, 2 and 5 μM (**Supplementary Figures S1A, B**). To ensure the specificity of TLK2 targeting, we examined TLK1 expression and observed a slight increase in TLK1 levels, which may reflect a compensatory response to TLK2 reduction (**Supplementary Figure S1C**). TLK2 ASO reduced cell viability in a dose-dependent manner in MOLM-14 and MONOMAC-1 cells (**Figure 1A**). Next, we assessed whether TLK2 ASO reduces viability in combination with a FLT3 inhibitor, gilteritinib. We observed a significant reduction in viability (up to 95%) with TLK2 ASO in combination with gilteritinib, in a dose-dependent manner, compared to NT ASO (**Figure 1B**). Gilteritinib IC_50s_ without ASOs in MOLM-14 and MONOMAC-1 are 9.18 nM and 18.84 nM, respectively (**Supplementary Figure S2A**). Addition of TLK2 ASOs decreased the gilteritinib IC_50_ in a dose-dependent manner in both cell lines. Specifically, MOLM-14 cells, the IC_50_ for gilteritinib with 1 µM, 2 µM, or 5 µM TLK2 ASO are 6.48, 5.17, or 3.79 nM, respectively. In MONOMAC-1 cells, the IC_50_ for gilteritinib with 1 µM, 2 µM, or 5 µM TLK2 ASO are 15.12, 12.20, or 9.24 nM, respectively (**Figure 1B**). To evaluate the synergies between TLK2 ASOs and gilteritinib, we ran Loewe analysis using Combenefit. We observed that TLK2 ASO showed synergy with gilteritinib in MOLM-14 cells and MONOMAC-1 cells (**Supplementary Figure S2B**). The highest synergy in MOLM-14 cells was observed with 5 µM TLK2 ASO and 15 nM gilteritinib (synergy score=16). In MONOMAC-1 cells, 5 µM TLK2 ASO combined with either 5 or 10 nM gilteritinib showed the strongest synergy (synergy score=10). This lack of a dose-dependent increase in synergy in MONOMAC-1 cells may reflect stronger dose-dependent efficacy of TLK2 ASO as a single agent, whereas gilteritinib plateaus at 50% efficacy at 5–10 nM, providing limited additional benefit at higher concentrations. Consistent with this, using SynergyFinder, the ZIP synergy score was higher in MOLM-14 cells (synergy score=7.587), but in MONOMAC-1 higher synergy is observed with only lower gilteritinib doses (**Supplementary Figure S2C**). However, HSA model also showed strong synergy between TLK2 ASOs with gilteritinib, with synergy scores 13.454 in MOLM-14 and 12.857 in MONOMAC-1 cells (**Supplementary Figure S2D**). Overall, gilteritinib and the TLK2 ASO show synergy at varying concentrations across different cell lines. Interestingly, gilteritinib showed limited efficacy in MONOMAC-1 cells, which could be related to the presence of p53 mutation (27), while the TLK2 ASO, as a single agent, reduced the viability of these cells, supporting the potential applicability of TLK2 ASO as a therapeutic strategy for heterogeneous AML.

Mechanistically, immunoblot analysis demonstrated that gilteritinib reduced p-FLT3 (Y591) and p-STAT5 (Y694) levels, while single treatment with TLK2 ASO reduced only TLK2 levels. However, TLK2 ASO in combination with gilteritinib further decreased p-FLT3 Y591 levels compared to either TLK2 ASO or gilteritinib alone treatments (**Figure 1C**), suggesting that the combination more effectively blocks the FLT3 pathway, and combined inhibition of TLK2 and FLT3 signaling may suppress AML cells’ growth more effectively compared to single treatments.

**Figure 2:**
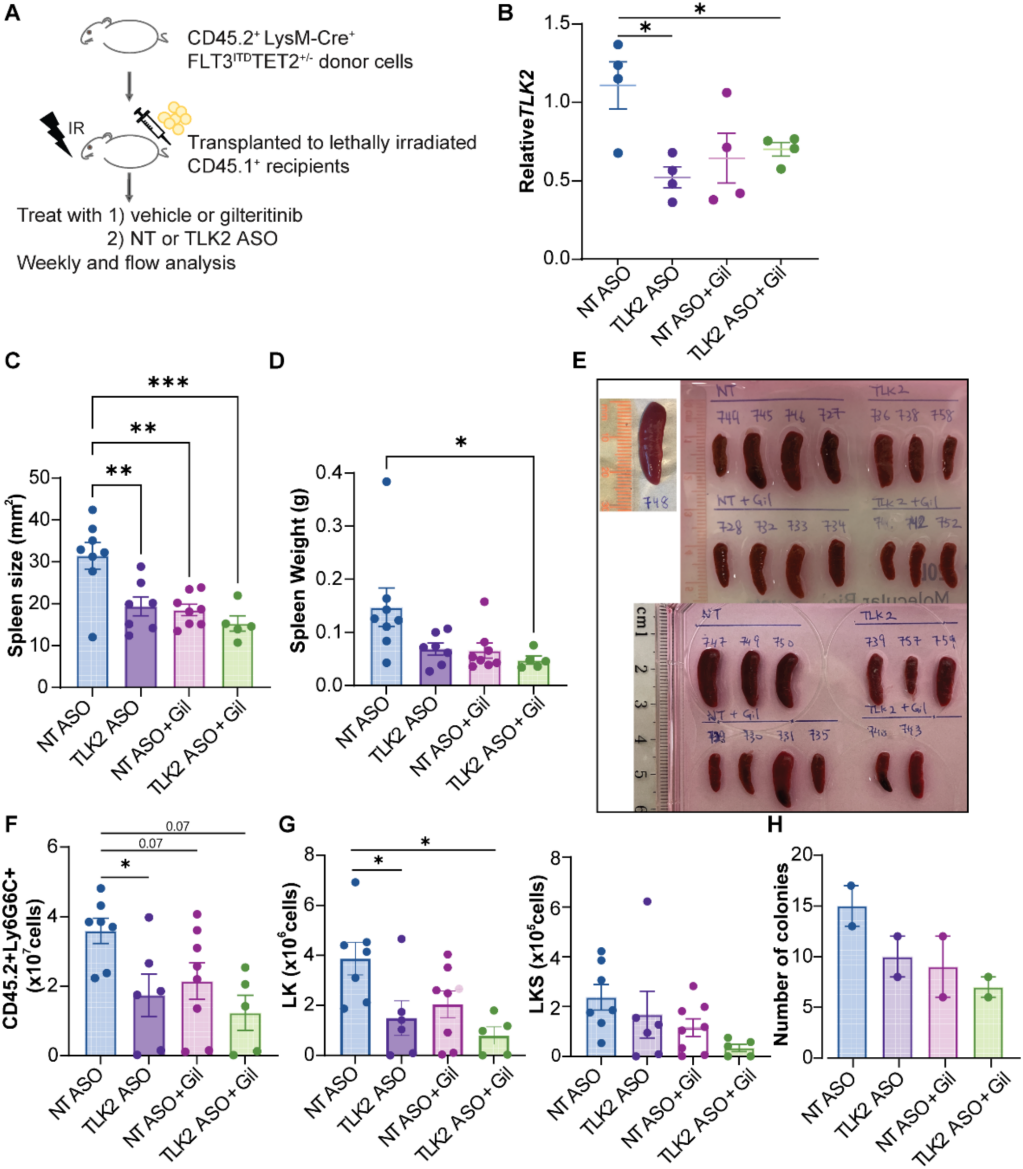
TLK2 ASO in combination with gilteritinib decreases leukemic burden in a murine model of AML. **(A)** A schematic representation of a murine AML bone marrow transplantation model. 1e6 lineage (Lin^-^)-depleted CD45.2^+^*LysM-Cre^+^Flt3^ITD^Tet2^+/-^* bone marrow cells were injected into lethally irradiated CD45.1^+^ recipients. Mice were treated with NT ASO, TLK2 ASO, NT ASO + gilteritinib, or TLK2 ASO + gilteritinib. **(B)** TLK2 expression in peripheral blood at week 17. **(C)** Spleen size measured by ultrasound at 14 weeks post-transplantation. **(D)** Endpoint spleen weights at 19 weeks post-transplantation. **(E)** Spleen images from each treatment group. **(F)** Leukemic burden in the bone marrow at 19 weeks post-transplantation was measured by flow cytometry using CD45.2^+^ and myeloid marker Ly6GLy6C. **(G)** Number of Lin^-^ c-Kit^+^ population and Lin^-^c^-^Kit^+^Sca^-^1^+^ population in the bone marrow at week 19. **(H)** 2, 500 lineage-negative bone marrow cells from *LysM-Cre^+^Flt3^ITD^Tet2^+/-^* mouse were plated in methylcellulose-based media and treated with 10 µM NT ASO or TLK2 ASO, either alone or in combination with 200 nM gilteritinib. Colonies were counted on day 6. Data was presented in duplicates. Statistical significance (*p < 0.05, **p < 0.01, ***p < 0.001) as determined by one-way ANOVA.

To assess the impact of ASO *in vivo*, we used a murine AML model where bone marrow chimeras were created using *LysM-Cre^+^Tet2^+/-^Flt3^ITD^* mice as donors and BoyJ CD45.1 mice as recipients. After engraftment confirmation, recipient mice were treated with NT or TLK2 ASO in combination with vehicle or gilteritinib (n= 5–8 mice per group) for 18 weeks. From 14 days post-bone marrow transplant until week 6, each recipient mouse was treated daily with 25 mg/ml gilteritinib orally and 30 mpk/mouse of either NT ASO (792169) or mouse TLK2 ASO (1545612-4) subcutaneously (210 mpk/mouse weekly). From week 7 to 13, 30 mg/ml gilteritinib and either 30 mpk/mouse of NT or TLK2 ASO daily (210 mpk/mouse weekly). From week 14 to 18, 30 mg/ml gilteritinib and 50 mpk/mouse of either NT or TLK2 ASOs 5 days per week (250 mpk/mouse weekly) (**Figure 2A**). Leukemia burden was assessed at the endpoint in the bone marrow and spleen. We observed 50% knockdown efficiency in peripheral blood at week 17 (**Figure 2B**). At week 14, ultrasound analysis was used to measure 2D spleen images and calculate the spleen size (**Supplementary Figure S3**). We found that the spleens of TLK2 ASO, gilteritinib, TLK2 ASO + gilteritinib-treated groups were 38%, 41%, and 52% smaller than NT-treated group (p = 0.006, p = 0.002, p = 0.001, respectively, **Figure 2C**). At the endpoint, TLK2 ASO + gilteritinib-treated group showed the smallest spleen weight, with a 58% reduction compared to the NT ASO group (p = 0.02, **Figures 2D, E**). We observed reduced 58% leukemia burden in BM of the TLK2 ASO group (p = 0.04, **Figure 2F**) and reduced 48% and 54% leukemia burden in gilteritinib group and combination groups at the endpoint (p = 0.07 and p = 0.07 respectively, **Figure 2F**). Lineage-negative and c-Kit^+^ (LK) populations were reduced in BM of both the TLK2 ASO alone and combination groups (p = 0.05, p=0.01, respectively, **Figure 2G**). Lineage negative, cKit^+^ and Sca-1^+^ (LKS) populations also showed a reduction trend in the combination group (**Figure 2G**), suggesting that TLK2 targeting may impair the survival of leukemic progenitor cells and reduce disease burden. Accordingly, the colony formation assays using lineage-negative cells derived from *LysM-Cre^+^Tet2^+/-^Flt3^ITD^* mice show that TLK2 ASO or gilteritinib alone reduced colony formation ability compared to NT ASO. The combination of TLK2 ASO and gilteritinib is more effective at reducing colony forming ability than either single treatment alone (**Figure 2H**). Slight weight loss was observed after week 15 (**Supplementary Figure S4A**), indicating the need for future optimization of the treatment strategy to minimize this effect. To assess the safety profile of TLK2 ASO, plasma was collected from mice treated with NT ASO, TLK2 ASO, gilteritinib, or TLK2 ASO and gilteritinib combination. Levels of ALT, AST, blood urea nitrogen, bilirubin, and albumin were measured using Beckman Coulter chemistry analyzer AU480 following the manufacturer’s protocols at Ionis Pharmaceuticals. The toxicity study showed that ALT levels in the TLK2 ASO group were slightly elevated, but the combination treatment reduced ALT levels. Other enzymes remained within a 5-fold change compared to the NT ASO group, which is considered tolerable (**Supplementary Figure S4B**).

## Discussion

This study highlights the therapeutic potential of targeting Tousled-like kinase 2 (TLK2) in acute myeloid leukemia (AML), particularly in patients with FLT3 mutations. Our findings demonstrate that antisense oligonucleotide (ASO)-mediated suppression of TLK2 results in a significant reduction in TLK2 mRNA levels, decreased leukemic cell viability and leukemia burden, and enhanced antileukemic activity when combined with the FLT3 inhibitor gilteritinib.

TLK2 is increasingly recognized as a regulator of DNA replication and chromatin remodeling, with recent studies implicating its dysregulation in various malignancies, including AML, breast cancer, lung cancer, and glioblastoma (5, 9, 10, 28). Despite its oncogenic associations, no selective inhibitors for TLK2 have been clinically developed, and available small-molecule inhibitors may affect the activity of TLK1 or other kinases (10, 12). In contrast, ASOs can be designed to selectively degrade TLK2 transcripts with minimal effects on TLK1 expression, making them an attractive therapeutic modality with reduced off-target activity.

TLK1 and TLK2 are highly homologous, therefore TLK1 may compensate for the function of TLK2 (5). Several phenothiazine compounds, including Thioridazine and J54, were identified as TLK1 inhibitors in a screen, though their effectiveness is limited and associated with toxicity (29–31) in prostate cancer cells. Thus, insights from this work may inform the design of ASO-based strategies against TLK1 that can also be used as a combined targeting of both TLKs.

The synergistic effect observed with TLK2 ASO and gilteritinib in FLT3-mutant AML cell lines is particularly promising. FLT3 mutations are among the most common and aggressive alterations in AML and are associated with poor prognosis (32). Gilteritinib, a selective FLT3 inhibitor, has shown efficacy in relapsed/refractory AML (33); however, resistance often emerges due to additional acquisition of mutations. Our data using two independent AML cell lines and a mouse model suggest that TLK2 inhibition can potentiate FLT3-targeted therapy, potentially overcoming therapeutic resistance and enhancing treatment efficacy. The *in vivo* results further support the translational potential of TLK2 ASO.

As a cautionary note, the efficacy of ASOs can be limited by their stability and delivery methods in different cell types. Future studies could focus on improving both the stability and delivery efficiency of TLK2 ASOs to achieve more robust target suppression *in vivo*. Although our current *in vivo* model achieved ~50% TLK2 knockdown, our published studies (5) indicate that deeper suppression may be required to improve the survival benefit observed with homozygous TLK2 deletion compared to heterozygous mice. Emerging lipid nanovesicle (LNV)–based delivery systems represent a promising strategy to enhance ASO protection from nucleases, extend half-life, and improve targeted uptake (34). Systematic optimization of LNV formulations for TLK2 ASOs, along with comprehensive evaluation of biodistribution, pharmacokinetics, and safety, could improve their efficacy.

Additionally, TLK2 is broadly expressed across AML genetic subtypes (5). Our data support that TLK2 ASO was able to suppress leukemic cell growth in various genetic subtypes and support the rationale for applying TLK2 ASO in combination with other AML-targeted therapies, such as venetoclax and azacitidine or Menin inhibitors (35, 36). This may help overcome molecular heterogeneity and provide therapeutic benefit for patients who relapse or fail to respond to current standard-of-care regimens. Although dual TLK1/TLK2 knockdown did not produce additive effects *in vitro (*5), the potential compensatory role of TLK1 *in vivo* is possible, where the tumor microenvironment and pharmacologic dynamics may influence therapeutic response. Therefore, future studies using TLK1/TLK2 double-knockout models could determine whether combined inhibition provides additional suppression of AML progression beyond TLK2-targeted strategies alone. Finally, expanding evaluation of TLK2 ASOs to additional hematologic and solid tumor contexts will clarify the broader oncologic applicability of TLK-directed therapy. Overall, optimization of ASO delivery, rational therapeutic combinations, and validation in clinically relevant preclinical models will help toward translating TLK2-targeted strategies into clinical testing.

## Data Availability

The original contributions presented in the study are included in the article/**Supplementary Material**. Further inquiries can be directed to the corresponding author.
