# Supplementary figures and images for: Targeting TLK2 with antisense oligonucleotides as a new strategy in acute myeloid leukemia
Source: Front Oncol. 2026 Mar 9;16:1659341. doi: 10.3389/fonc.2026.1659341 (PMC13006219; doi:10.3389/fonc.2026.1659341)

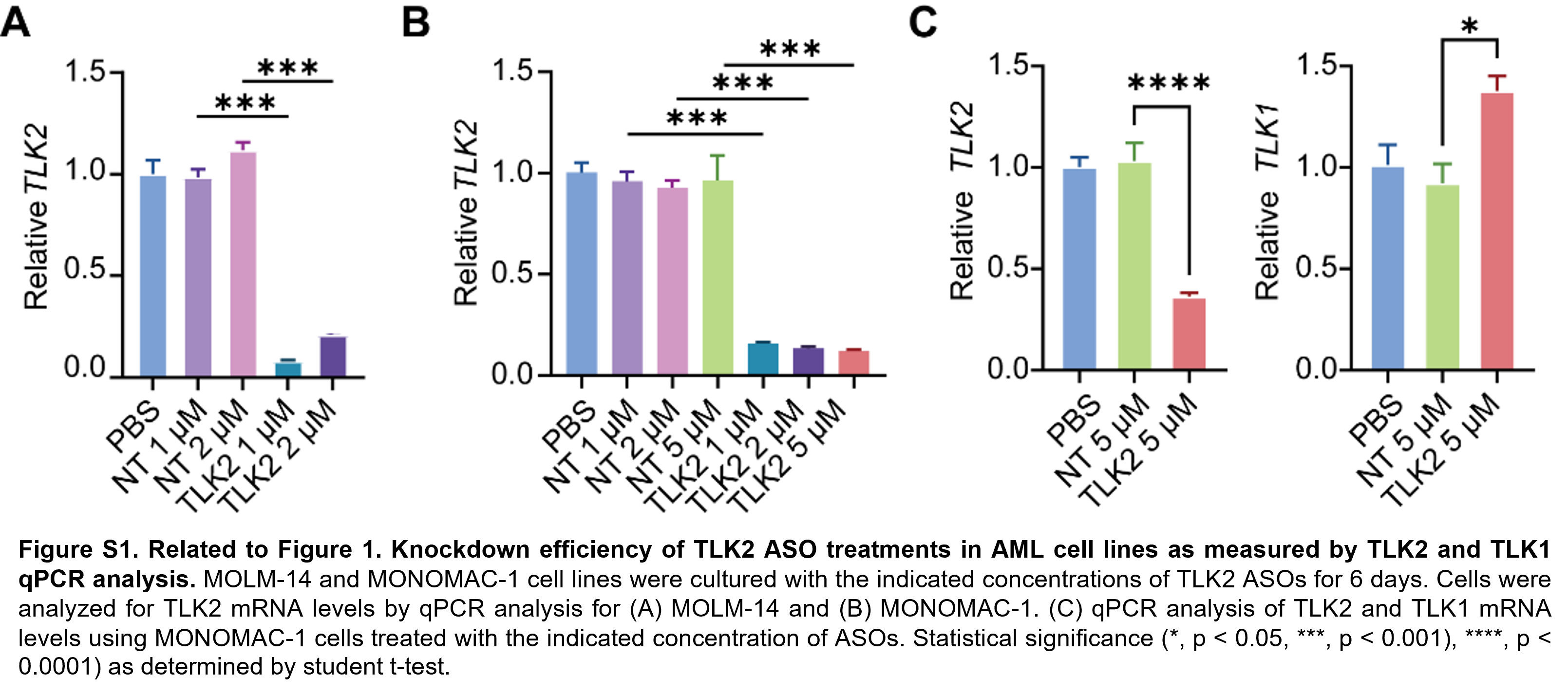

Supplement: Supplementary file 1 [file Image1.png]

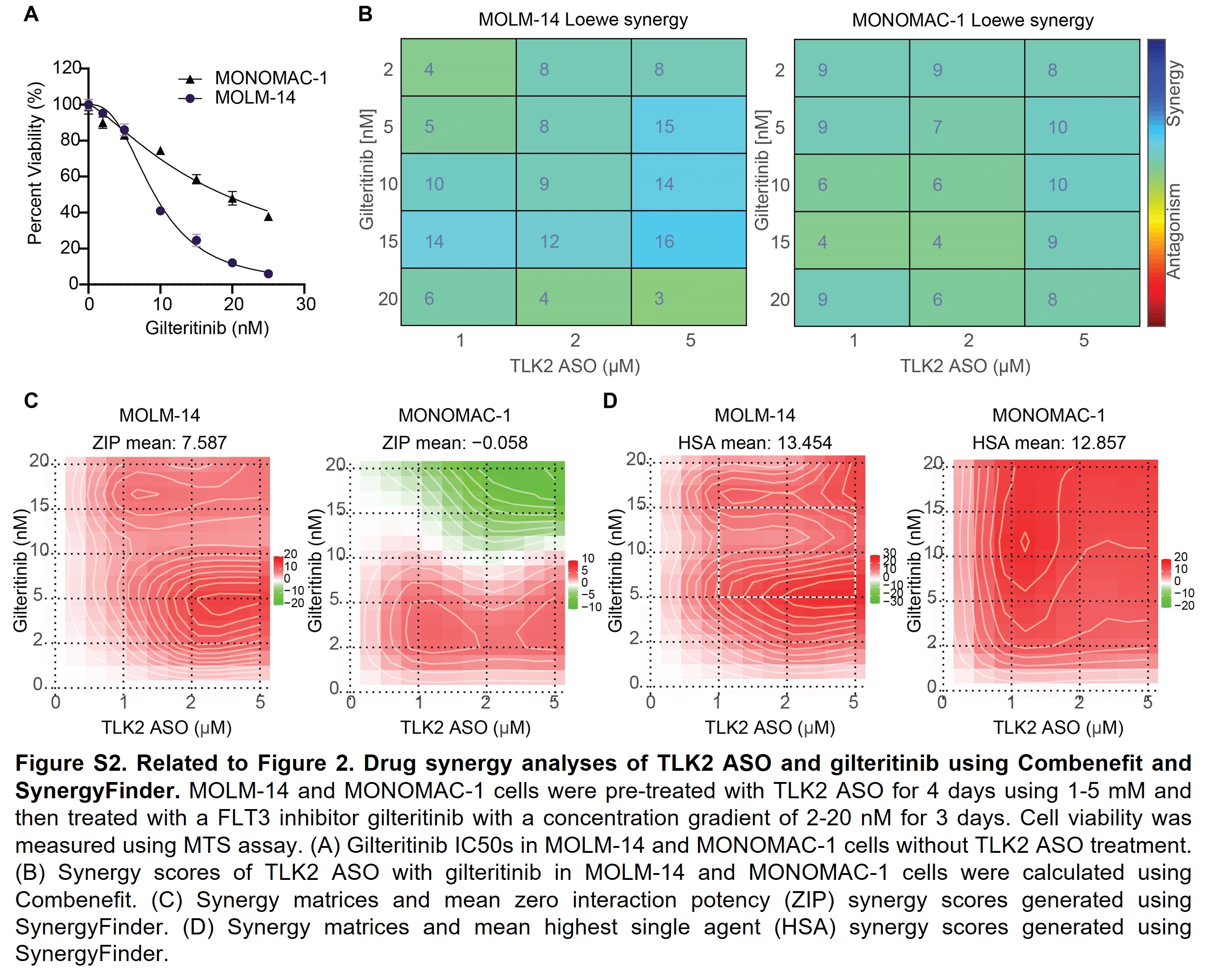

Supplement: Supplementary file 2 [file Image2.jpg]

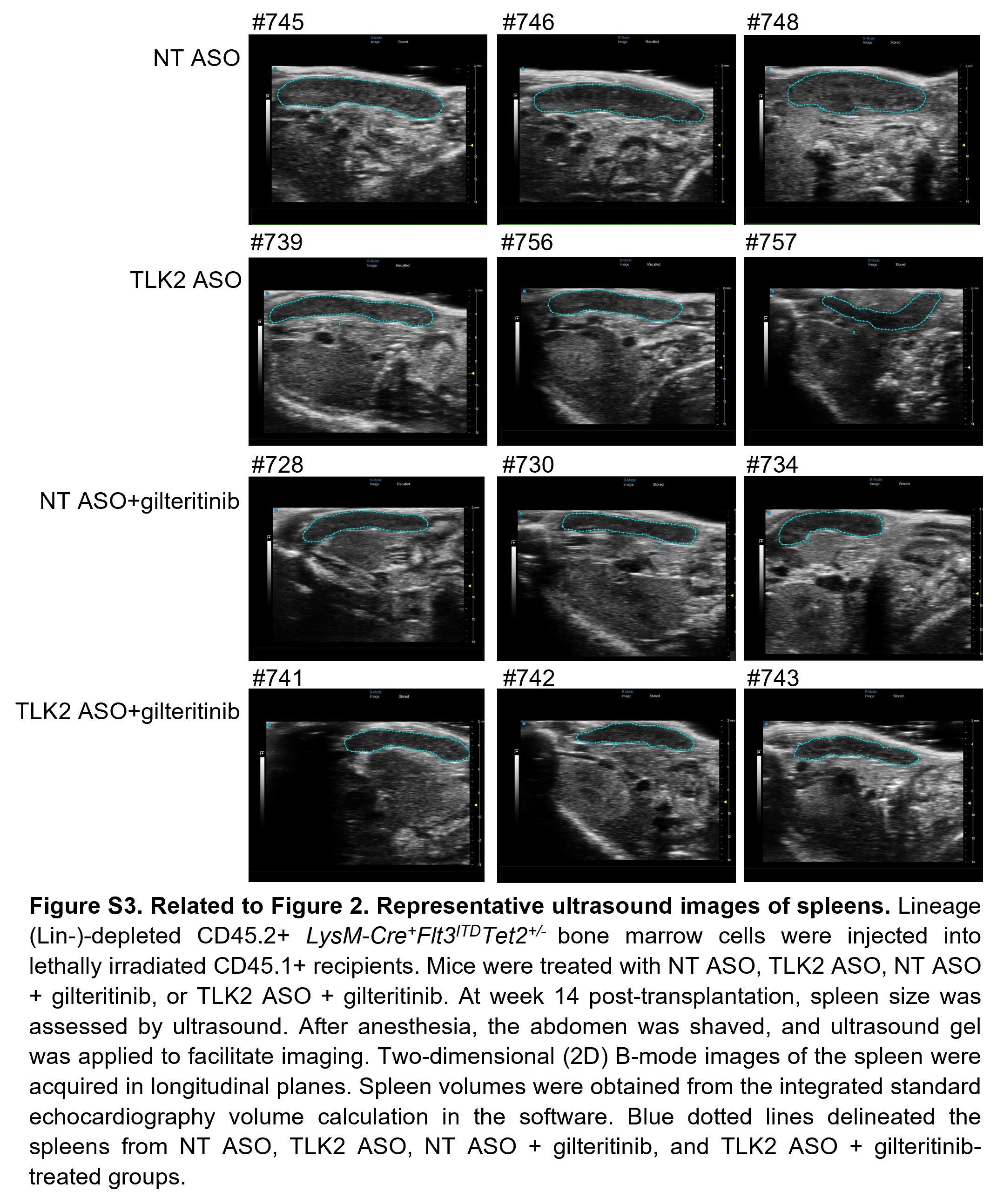

Supplement: Supplementary file 3 [file Image3.png]

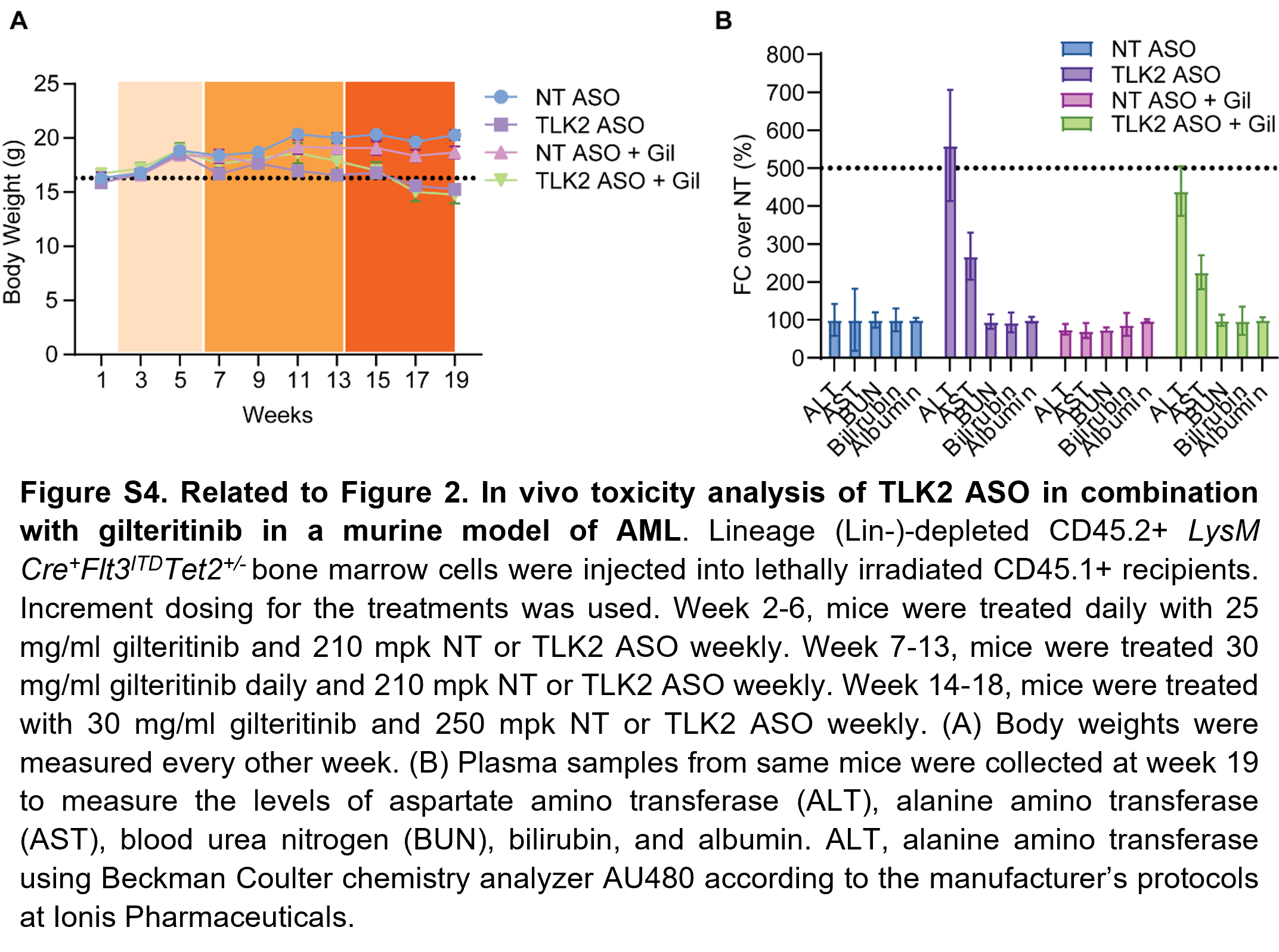

Supplement: Supplementary file 4 [file Image4.png]
